# Preface: a new stage in the evolution of public health reviews

**DOI:** 10.1186/s40985-015-0003-2

**Published:** 2015-05-29

**Authors:** Laurent Chambaud, Theodore Tulchinsky

**Affiliations:** 1 Dean of EHESP, Paris and Rennes, France; 2 Braun School of Public Health, Hebrew University-Hadassah, Jerusalem, Israel; 3 Ashkelon College Ashkelon, Ashkelon, Israel

## Foundation and Continuation of *Public Health Reviews*

*Public Health Reviews (*PHR) was founded in 1972 in Israel and published for over 30 years, with listing in Pubmed, Medline and Index Medicus and many other on-line data bases until the owner retired. The journal was purchased in 2010 by the EHESP (*Ecole des Hautes Études en Santé Publique, the* French National School of public health) and established with a distinguished international Editorial Board.

The mission of PHR since 2010 has been to provide access to European and global public health students, teachers, researchers, practitioners and policy makers of theme topic reviews of high quality to promote learning and discussion of issues in public health from a multi disciplinary point of view. The Editorial Board participated actively in policy development and leadership of many of the theme issues that evolved. PHR strives to bring topical reviews to promote translation of evidence and science into applied public health policy and practice, including the evolution of health systems as part of public health spectrum [1].

The theme topics PHR issues during 2010–2013 included:

- The New Public Health
- Ageing Societies
- Public Health Education
- Cardiovascular Disease
- Ethics in Public Health
- Mental Health as a Public Health Issue
- Towards a Healthier 2020, and
- Substance Use Issues: New Insights.

## The New Home for Public Health Reviews 2014

In 2014, a further reformation of PHR was arranged with affiliation and sponsorship by the Association of Schools of Public Health in the European Region (ASPHER), along with continued sponsorship by EHESP, and an additional sponsor, a Swiss School of public health. An agreement was signed with Biomed Central to publish PHR as an online open access journal. The new arrangements for sponsorship and operation of PHR provided for the continued mission of “each issue addressing concepts such as health disparities, social determinants of health, demographic shifts and changing patterns of health needs, education of the public health workforce and policy makers, and healthcare delivery systems.” [1].

A new Editorial Board (EB) was organized with a strengthened European, international and multi-disciplinary composition to continue the excellent work of the previous EB. A core group of ASPHER representatives working with the editorial team provided important support over the transition period. The previous policies of theme issues will be augmented by accepting individually submitted review papers which fit in with topical issues. PHR will strive to achieve monthly issues, yielding 30 articles annually, with free for online access, including archived previous volumes.

## The Opening Issue of PHR in 2015

The initial issue is a follow up to the 2012 volume on public health ethics. A second issue of PHR on this theme within four years of a previous issue is based on the initiative of ASPHER to prepare curriculum material for formal and informal teaching of public health ethics in the European context as explained in the Lee and Royo-Bordonada editorial [2], and Towards Public Health Ethics [3]. A European oriented curriculum will need relevant case studies for the organized teaching of ethics in public health education programs for undergraduate and graduate degree programs in Europe, in parallel to current Canadian and U.S. initiatives in public health ethics case studies [4].

Public health has many enormous achievements to its credit adding many years of life expectancy globally. But public health has also suffered from past ethical failures of staggering proportions in the 20th century through association with eugenics. The most egregious of these was the active and passive participation of medical and public health professionals in interpretations of eugenics which led to mass sterilization and the murder of handicapped people, and participation in the Holocaust perpetrated by Nazi Germany. This took place and was enabled by governments, the medical and public health professions, business, professional and religious organizations, through active participation or silent acquiescence across Nazi occupied Europe [5]. From these horrific events emerged the Nuremberg Doctors Trials (1946), the Universal Declaration of Human Rights (1948), the United Nations Convention on the Prevention and Punishment of the Crime of Genocide (1948), the World Medical Association Declaration of Helsinki Ethical Principles for Medical Research Involving Human Subjects in1964 and revised regularly since.

These internationally accepted conventions set new global standards of the most basic ethical standards of ancient Biblical commandment of “thou shalt not kill” and ethical precept of the “Sanctity of Life”, along with the Hippocratic physician’s oath of “do no harm” [6].

Ethical considerations in public health are also relevant to the public discourse when evidence-based interventions are implemented or not implemented amid societal controversy. Neglect or failure to implement current best practices is of public concern and debate, for example in the current measles epidemic in Europe and particularly controversial in the United States when refusal of some parents to immunize their children putting other children at risk. Public health ethical issues most often occur in the interface of community rights and individual rights. But in societies based both on law and on protection of individual liberties, societies have obligations for health protection and prevention even if individual rights are legally restricted, such as driving on the wrong side of the road, without seat belts and while texting, or putting workers in dangerous mines, or refusing to immunize a child going to school which puts other children at risk [7].

The study of and competent discussion of public health ethics are essential components of education of health professionals and the practice of public health. New challenges are evolving rapidly and on a continuing basis, such as quarantining persons exposed to Ebola or micronutrient fortification of foods and supplements for groups with or vulnerable to important deficiency conditions. Increasing societal challenges, such as obesity, diabetes, palliative care, end of life medical help, with adoption of new technologies and evidence based measures of preventive medicine and health promotion involving individual and community reposnoibilities.

Ethical issues in public health do not revolve solely around individual liberties versus government controls [8]. Public health involves many stakeholders and actors. Governments, local authorities, advocacy groups, insurance systems, professional associations, religious organizations, social services, academic teaching and research centers, food and drug manufacturers are all contributors or actors in what is increasingly being called “The New Public Health” [9].

The world struggles to reduce poverty, racism, terrorism, and health inequities, improving equal rights for all persons, and protection of human rights and the environment, are the “uber issues” of public health that all involve the development of more equal societies and global health. There are many public health methodologies and practices involved in these issues all requiring professional awareness and sensitivity to ethical issues. At the same time public health has an advocacy role for promoting adoption of new scientific advances and timely adoption of documented successful “best practices” and “evidence-based” methods of improving population health. Reducing health inequalities, development of universal health coverage, access to primary and integrated care, improving maternal and child health and application of risk reduction measures for non communicable diseases are all crucial global health issues.

Public health has a challenging future which requires building public credibility in face of opposition and reluctance to immunize or to adopt healthy lifestyles, unjustified delayed adoption of effective prevention methods, and challenges and rollbacks due to anti-public health activism. PHR will continue to address public health ethics in the future with case studies and teaching methods to ensure that present and future public health teachers, practitioners and policy makers have the competencies needed to analyze and address current and future challenges of modern public health.

## Our Outlook for PHR

The challenges of public health range widely across the whole health sector from health promotion and disease prevention, health care systems development, long term and terminal care as well as nutrition security, climate change, social inequality and many others. PHR’s mission is to strengthen a multi- and interdisciplinary perspective to better understand the new challenges in public health. We are developing theme issues on screening; climate and health; minority, migrant and aboriginal health; ageing; maternal and child health; human rights and health. Other topics will be generated by the Editorial Board to address the greatest causes of inequality of mortality, disease and disability including poverty, ignorance, societal oppression of women as well as the special needs of high risk groups.

PHR will continue its tradition of public health advocacy and promoting adoption of best practices and evidence based standards of scientific knowledge for our target audiences in the European region and globally. We are embarking on a new path organizationally and hope to continue and improve our quality and outreach to enhance our mission to advance public health awareness and practice globally.
